# Role of Base Grease Type on the Lubrication Performance of Hexagonal Boron Nitride Nanoparticles and Microparticles

**DOI:** 10.3390/ma18102196

**Published:** 2025-05-09

**Authors:** Szymon Senyk, Krzysztof Gocman, Marcin Wachowski, Tadeusz Kałdoński

**Affiliations:** Faculty of Mechanical Engineering, Military University of Technology, 00-908 Warsaw, Poland; krzysztof.gocman@wat.edu.pl (K.G.); marcin.wachowski@wat.edu.pl (M.W.); tadeusz.kaldonski@wat.edu.pl (T.K.)

**Keywords:** greases, hexagonal boron nitride, nanoparticles, microparticles

## Abstract

This study investigates the influence of hexagonal boron nitride (h-BN) particle size and concentration on the tribological performance of lithium and calcium greases. Formulations containing h-BN nanoparticles and microparticles at 1%, 3%, 5%, and 10% by weight were evaluated in ball-on-flat reciprocating tests under three load conditions. The tests were conducted using a steel ball and a steel plate. The most favorable results were obtained for greases with 3% h-BN, characterized by an average particle size of 130 nm and the highest nanoparticle content. In lithium grease, this formulation reduced friction by up to 9.7% and wear by up to 69.2% compared to the base grease. In calcium grease, the same additive concentration led to reductions of up to 18.2% in friction and 70.2% in wear. Tribological performance was significantly influenced by the type of base grease, which affected the dispersion of the additive and its ability to form protective surface layers. SEM/EDS analysis of the surfaces after testing revealed that the dominant lubrication mechanisms included shearing-sliding and surface-mending effects. This study confirms that h-BN—especially in nanoparticle form—is an effective additive for improving the performance of greases.

## 1. Introduction

Greases constitute a category of lubricants widely used in tribological systems. Their application facilitates the separation of contacting frictional surfaces, transforming external friction between solids into internal friction within the lubricant. This phenomenon reduces frictional resistance between interacting components and significantly mitigates wear.

Lugt [1] highlighted the challenges in grease research, including the development of greases capable of providing longer service life for lubricated components and performing reliably under harsher operating conditions. This demand aligns with the vision for the advancement of tribology presented in 2017 by Holmberg and Erdemir [2]. Among the strategies for reducing frictional resistance and wear, they emphasized the importance of developing new lubricants and highlighted the role of anti-friction and anti-wear nano-additives. In light of these insights, further research is essential to tailor the properties of greases using modern additives that can enhance their tribological performance.

Solid lubricants offer a promising solution in this context. These materials are characterized by low internal friction and relatively weak cohesive forces, which enable their plastic deformation. Their behavior is comparable to that of liquid lubricants, as they can adhere to friction surfaces and form protective layers [3,4,5]. Hexagonal boron nitride (h-BN), a layered compound, is a prominent example of a solid lubricant. The rationale for its application has been extensively discussed in the literature [5,6,7,8,9,10,11,12,13,14].

A review of the literature on grease additives indicates that only a limited number of publications focus on hexagonal boron nitride. In the first study in this field [6], the h-BN used featured particles with an average size of approximately 0.5 µm. Adding this material to greases at concentrations of 3%, 5%, and 10% had a positive effect on their tribological properties. Subsequent studies [15,16,17,18,19] focused on searching for the optimal concentration of h-BN and confirmed its effectiveness; however, findings remain somewhat variable due to differences in particle size, shape, and base grease. A series of studies [20,21,22] concerned the use of hexagonal boron nitride at different concentrations and particle sizes as an additive in greases applied during aluminum forming processes. These studies concluded that h-BN could serve as an alternative to graphite in the analyzed processes, provided that its concentration and particle size are appropriately selected.

Since 2019, the number of studies on the use of various hexagonal boron nitride nanoparticles has increased [23,24,25,26,27]. These nanoparticles have consistently demonstrated their effectiveness as anti-friction and anti-wear additives, although they are typically used at a maximum concentration of 3% [27]. Given the diversity of particle sizes used and advancements in the field of nanomaterials, a logical approach would be to compare the effects of hexagonal boron nitride nanoparticles and microparticles on the tribological properties of greases. This concept was implemented in [28], where three types of h-BN were tested at a concentration of 4%. In this case, lubrication efficiency was strongly influenced by the particle size of the additive, with the grease containing h-BN nanoparticles exhibiting the best anti-friction and anti-wear properties. According to [28], the most favorable tribological properties of greases are obtained when the concentration of nanoparticles does not exceed 4%, as higher concentrations may reduce performance enhancement due to agglomeration issues. Other researchers [29] have investigated the tribological properties of greases containing both h-BN microparticles and nanoparticles, and confirmed that smaller particles, when used in appropriate concentrations, were particularly effective in enhancing anti-wear characteristics. Analyses of compounds analogous to h-BN, such as graphite [30] and molybdenum disulphide (MoS_2_) [31], yielded similar findings. The introduction of nanoparticles into greases has been shown to provide superior performance compared to larger particles.

Hexagonal boron nitride has been shown to positively influence the tribological properties of greases, with these properties being determined primarily by the concentration and granularity of the additive. To date, research has predominantly focused on the effect of additive concentration; however, the influence of the base grease type remains insufficiently studied, as most cited works involve only one type of base grease—typically lithium grease. Another underexplored aspect concerns the physicochemical properties of hexagonal boron nitride, such as particle morphology, size distribution, and porous structure. Although these factors are critical in determining the effectiveness of h-BN as an additive, they have received limited attention in the scientific literature. A thorough review of existing studies highlights the need for further research to evaluate the effects of varying concentrations of hexagonal boron nitride nanoparticles and microparticles on the tribological properties of different base greases.

The aim of this paper is to evaluate the tribological properties of lithium and calcium greases with hexagonal boron nitride at varying fractions of nanoparticles and microparticles and different concentrations. The experimental setup employs a ball-on-flat contact operating in reciprocating motion, allowing for the assessment of the greases’ anti-friction and anti-wear properties. Additionally, the effect of the UNMT tribometer friction node load (Section 2.2) on these properties is investigated.

## 2. Materials and Methods

### 2.1. Hexagonal Boron Nitride and Base Greases

In this study, four types of hexagonal boron nitride were used, the physical and chemical properties of which have been described in detail elsewhere [32]. The parameters characterizing the size of the hexagonal boron nitride particles and their porosities are presented in Table 1.

The additives were incorporated into two base greases at mass concentrations of 1%, 3%, 5%, and 10%. Lithium grease (L) was prepared from mineral oil thickened with lithium 12-hydroxystearate, while calcium grease (C) was prepared using mineral oil thickened with the reaction product of a mixture of fatty acids and calcium hydroxide. The consistency, thermal and mechanical stability, as well as the microstructure of the selected greases used in this study are described in [33]. A summary of the greases is presented in Table 2.

### 2.2. Tribological Test

**Figure 1:**
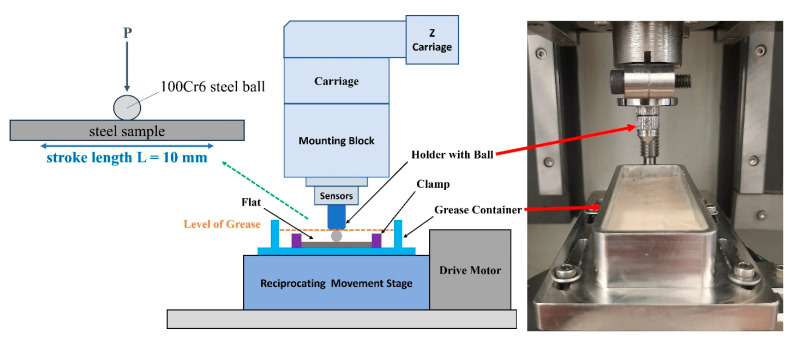
UNMT Tribotester with instrumentation for reciprocating motion testing.

Reciprocating tests were performed on a UNMT Tribotester (Universal Nano/Micro Tester, CETR, Campbell, CA, USA). The friction node used was a ball-on-flat system. A schematic diagram of the test rig is shown in Figure 1.

The plates were made of tool steel according to the standard [34]. The hardness of these plates was approximately 62 HRC, and the surface roughness, described by the Ra parameter, was equal to 0.01–0.02 µm. The counter samples were balls with a diameter of 3.2 mm, made of 100Cr6 (AISI 5210, 1.3505) bearing steel. The chemical composition of the steel grades used for the balls and plates is presented in Table 3.

The tribological test methodology was based on Procedure B presented in ASTM G133 [36]. Following the normative guidelines, a stroke length of 10 mm, an oscillation frequency of 10 Hz, and a test duration of 2000 s were used. Due to the available instrumentation, a ball with a diameter different from that specified in the standard was used—3.2 mm instead of 4.76 mm. This reduction in ball size resulted in a smaller contact area and higher local contact pressures, which may have intensified wear phenomena and enhanced the visibility of differences in the performance of the tested grease additives. The tests were carried out with loads of 50 N, 100 N, and 150 N. Due to the limitations of the available Tribotester, tests at elevated temperatures were not performed. Therefore, the tests were conducted at ambient temperature. It should be noted that the use of a short stroke and moderate pressure in the test configuration may promote boundary lubrication conditions. The test conditions are summarized in Table 4.

During the tests, both the friction force and the load force were recorded, and the coefficient of friction was calculated using a computer program. In addition to the curves of the coefficient of friction as a function of time, the average coefficient of friction was also recorded during the measurement.

**Figure 2:**
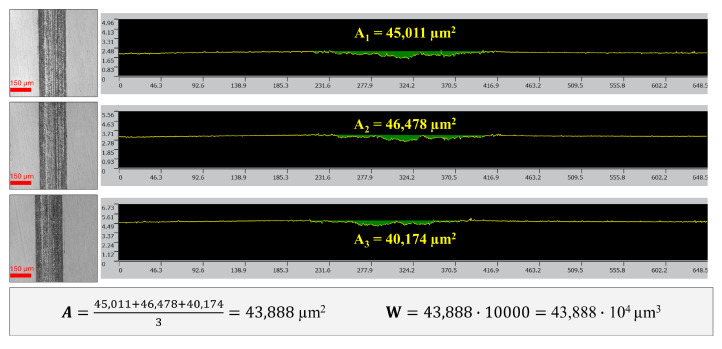
Wear measurement method (example for base lithium grease, 50 N load).

A method similar to ASTM G133 [36] was used to quantitatively assess the wear of the specimen. This method is similar to the method used in many publications, which involves measuring the cross-sectional area along the length of the sliding stroke [37]. Based on the provisions of the cited standard, the wear volume of the specimen (W) was expressed as the product of the average cross-sectional area (A) and the length of the sliding stroke (L), which was 10 mm in the described tests. For each wear trace, three profiles (A_1–3_) were recorded, each evenly distributed along the wear trace—one in the central wear area and two at the extremities at a distance of about 1 mm from the end of the trace. The rounded zones at the extremities of the trace were omitted from the analysis. An example of a wear measurement is shown in Figure 2. The measurements were taken using a LEXT OLS4100 3D Laser Scanning Confocal Microscope (Olympus, Tokyo, Japan), with an objective of 20× magnification.

The coefficient of friction and wear results presented in this article represent the arithmetic average of these parameters, calculated from the three tests performed.

In order to identify the interaction mechanisms of hexagonal boron nitride dispersed in the greases used to lubricate the components of the friction system under study, microscopic studies were performed for selected greases. Surface imaging after the friction process and microanalysis of the chemical composition of the steel substrate were carried out. The tests were conducted using a Zeiss Ultra Plus scanning electron microscope (Zeiss AG, Oberkochen, Germany), equipped with a Bruker Quantax 400 EDS microanalysis system (Bruker, Billerica, MA, USA). The images were acquired at an accelerating voltage of 3 kV, while the microanalysis of the chemical composition was conducted at 15 kV. The steel samples were cleaned with acetone after the tribological tests and prior to the microscopic observations.

## 3. Results

**Figure 3:**
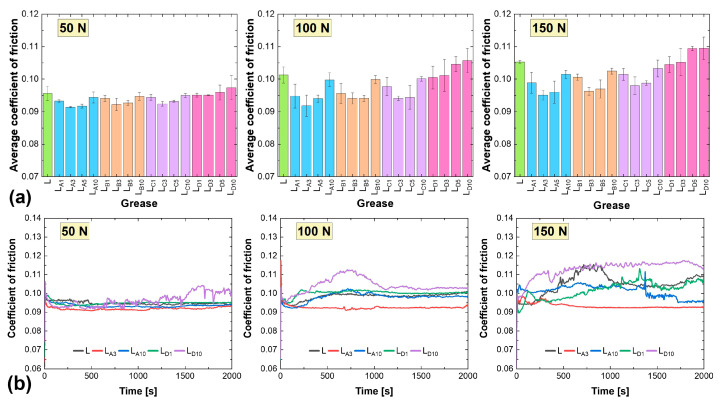
Friction properties of lithium greases: (**a**) average coefficient of friction, (**b**) examples of coefficient of friction curves.

The average values of coefficient of friction obtained for the lithium greases are presented in Figure 3a. The lowest values for each sample were recorded at a load of 50 N, while the highest occurred at 150 N. For the base sample, the coefficient of friction was 0.0956 at 50 N, 0.1013 at 100 N, and 0.1053 at 150 N. At a load of 150 N, symptoms of lubricant film instability were observed, as evidenced by pronounced fluctuations in the coefficient of friction (Figure 3b). Notably, larger average values at successive loads were observed only in samples containing additive D at concentrations of 5% and 10%. These increments reached a maximum of 1.9% at 50 N, 4.3% at 100 N, and 4.0% at 150 N. At a high concentration (10%) of this hexagonal boron nitride grade, the lubricating film exhibited instability, leading to significant variations in the coefficient of friction during testing, regardless of the applied load (Figure 3b). Conversely, when smaller amounts of this additive were present in the friction zone—specifically at concentrations of 1% and 3%—the recorded average coefficient of friction was comparable to those of the base composition. Lubrication with the L_D1_ composition demonstrated greater stability in the coefficient of friction over the test period compared to the L_D10_ grease, particularly under loads of 50 N and 100 N (Figure 3b).

Better tribological test results in terms of frictional resistance at the ball-on-flat contact were obtained for lithium greases containing additives A, B, and C, with additive A demonstrating particularly favorable performance. The coefficient of friction for lithium greases containing hexagonal boron nitride coming from Sample A at concentrations of 3% and 5% exhibited minimal variation with increasing load. For the 3% concentration, the values ranged from 0.0914 to 0.0951, while for the 5% concentration, they ranged from 0.0917 to 0.0960. As a result, the L_A3_ composition achieved the lowest average coefficient of friction among the tested lithium greases. Compared to the base grease, lubrication with this composition at successively applied loads resulted in reductions of 4.4%, 9.4%, and 9.7%. This demonstrates that the optimal concentration of additive A ensured highly effective lubrication, as evidenced by the stability of the coefficient of friction across varying loads (Figure 3b). Higher average values were observed for greases containing 1% and 10% additive A. At a 1% concentration, the particle density in the friction zone appears to have been insufficient to provide adequate surface protection and minimize frictional resistance. In contrast, at a 10% concentration, excessive particle agglomeration likely occurred. These clusters may have disrupted the uniform distribution of the grease within the friction zone, as evidenced by the curves shown in Figure 3b.

The results of tribological tests for lithium greases containing additives B and C were comparable. The average coefficient of friction ranged from 0.0922 to 0.0950 at a load of 50 N, from 0.0941 to 0.1001 at 100 N, and from 0.0963 to 0.1033 at 150 N. This similarity was likely due to the comparable physical and chemical properties of these types of hexagonal boron nitride. The conclusions regarding the optimal concentration of these additives, from an anti-friction perspective, were consistent with those drawn for additive A. The use of extreme concentrations (1% and 10%) resulted in a higher coefficient of friction values compared to intermediate concentrations. However, in a direct comparison of tribological performance, additive B, characterized by a slightly smaller particle size (with a higher proportion of nanoparticles) than additive C, exhibited superior performance. Additionally, the porous structure of additive B particles was more developed, as indicated by a 10% larger specific surface area and a 25% greater total pore volume compared to additive C (Table 1).

**Figure 4:**
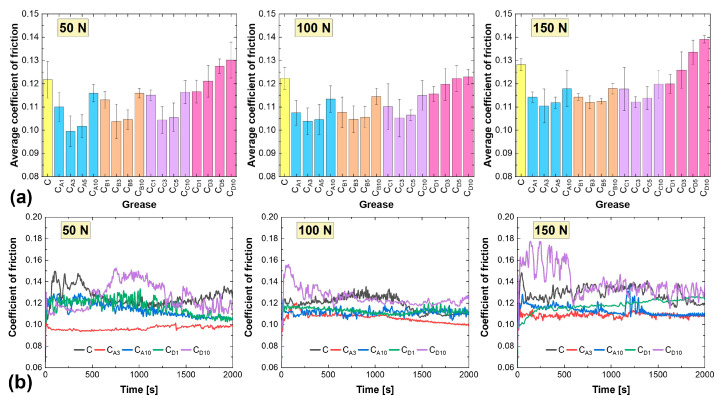
Friction properties of calcium greases: (**a**) average coefficient of friction, (**b**) examples of coefficient of friction curves.

The average coefficients of friction obtained for calcium greases are shown in Figure 4a, and these values were higher compared to those of the lithium greases. When lubricated with the base calcium grease, the coefficient of friction was 0.1217, 0.1223, and 0.1282 at progressively higher loads. This can be attributed to the more compact consistency of the base calcium grease (NLGI class 4) compared to the base lithium grease (NLGI class 2), which was investigated in [33]. As a result, the rheological characteristics of the calcium grease were associated with lower fluidity, leading to higher frictional resistance at the ball-on-flat contact. This also resulted in less stable frictional resistance compared to the base lithium grease, particularly at 50 N and 100 N loads (Figure 4b).

For ten compositions, including all those containing additive D and Samples C_A1_, C_A10_, C_B1_, C_B10_, C_C1_, and C_C10_, the smallest average coefficient of friction was recorded at a load of 100 N. In the case of the hexagonal boron nitride of Sample D, this phenomenon was attributed to a large particle size (Table 1). For compositions containing additives A, B, and C, this effect was due to either too low (1%) or too high (10%) particle concentrations. Most importantly, the low fluidity and highly compact structure of calcium grease made the selected hexagonal boron nitride particles less effective at penetrating the friction micro-regions during reciprocating sliding motion. When a load of 100 N was applied, the calcium greases—subjected to increased mechanical stress—became more fluid and more effectively reached the contact surfaces. As a result, the distribution of h-BN particles in the friction zone was more efficient. When the load was increased to 150 N, an increase in the average coefficient of friction was observed (Figure 4a). This trend was consistent for all calcium greases.

In the group of calcium greases containing hexagonal boron nitride from Sample D, two compositions, namely C_D5_ and C_D10_, exhibited a higher (or, in one case, equal) average coefficient of friction compared to the base composition. The changes in these values were a maximum of 7.0%, 0.5%, and 8.5% at progressively increasing loads. At lower concentrations of this additive, an improvement in the anti-friction properties of the calcium grease was observed. This was reflected in lower average values and greater stability during the test, as demonstrated by the comparison of the C_D1_ and C_D10_ compositions in Figure 4b.

The best results in the reciprocating tribological tests under the discussed conditions were obtained for the calcium greases containing additive A; the coefficient of friction at the ball-on-flat contact ranged from 0.0955 to 0.1159 at a load of 50 N, from 0.1039 to 0.1134 at 100 N, and from 0.1104 to 0.1178 at 150 N. The best anti-friction properties were observed for the C_A3_ grease. The dispersion of the selected particles in the calcium grease contributed to the stabilization of frictional resistance compared to the base calcium grease (Figure 4b). This resulted in a reduction of the average coefficient of friction by 18.2% at 50 N, 15.0% at 100 N, and 13.9% at 150 N. As with the lithium greases, the use of additive A at too high a concentration (10%) led to an increase in frictional resistance in the lubricated contact (Figure 4b). This was likely due to the agglomeration of these particles, which would impede the flow of grease into the friction zone.

The second most effective anti-friction additive introduced was hexagonal boron nitride, designated as B. The lowest coefficient of friction was recorded for a sample containing 3% of this additive. Slightly inferior results were obtained with additive C. The relationships with respect to h-BN concentrations were similar to those observed for the additive A and B. Extreme concentrations (1% and 10%) performed worse than the intermediate concentrations, resulting in higher coefficients of friction. The best results among the lubricants containing hexagonal boron nitride from group C were achieved at a concentration of 3%.

**Figure 5:**
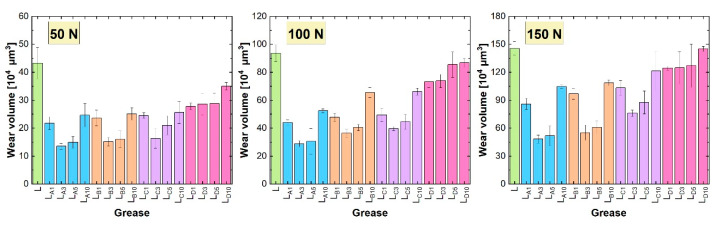
Wear of steel plates lubricated with lithium greases.

Figure 5 shows the average wear volume values recorded on the plate when lubricated with lithium greases. The wear recorded for the base grease L was 43.263 × 10^4^ µm^3^, 93.586 × 10^4^ µm^3^, and 145.669 × 10^4^ µm^3^ at progressively increasing loads. Wear decreased when lubricated with compositions containing hexagonal boron nitride. Compared to the base sample, the changes ranged from 19.1% to 68.7% at 50 N, from 7.4% to 69.2% at 100 N, and from 0.4% to 66.9% at 150 N.

The lowest wear values were achieved with the L_A3_ grease, with values of 13.547 × 10^4^ µm^3^, 28.808 × 10^4^ µm^3^, and 48.187 × 10^4^ µm^3^. The anti-wear effect of hexagonal boron nitride particles dispersed in the L_A3_ grease was linked to the smallest average particle size of this additive, which was 130 nm, and the highest proportion of nanoparticle fractions (Table 1). These particles also exhibited a developed porous structure. Therefore, it is likely that they effectively penetrated the friction zone along with the grease, settling in micro-regions and thereby protecting the cooperating surfaces from wear. Slightly higher wear was recorded with the L_A5_ composition. Similarly, as with the anti-friction performance, the compositions containing 1% and 10% of additive A performed worse than these two greases. The reasons for the lower anti-wear effectiveness of these h-BN concentrations were similar to those discussed regarding the frictional resistance results.

The anti-wear performance of lithium greases with the same content of additives B and C was similar; wear on the test plate at successive loads for these additives ranged from 15.161 to 25.632 × 10^4^ µm^3^, from 36.304 to 66.108 × 10^4^ µm^3^, and from 55.067 to 121.271 × 10^4^ µm^3^. This similarity was again related to the comparable physicochemical properties of these types of hexagonal boron nitride. The effectiveness ranking of the different h-BN concentrations was the same as that for the anti-friction properties.

Despite their minimal impact on anti-friction properties, additive D particles protected the friction surfaces from wear more effectively than the base composition, as indicated by the lower wear values recorded on the test plate. The lower the concentration of these particles, the better the protection of the surface against wear. This was consistent with the results of the average coefficient of friction measured during lubrication with these compositions.

**Figure 6:**
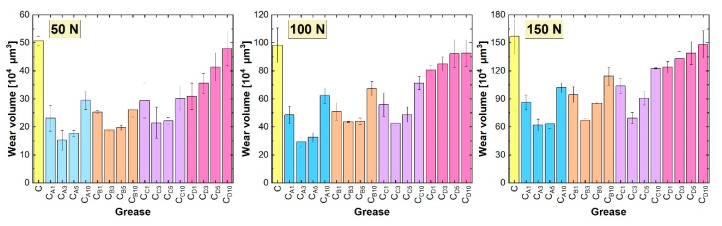
Wear of steel plates lubricated with calcium greases.

Figure 6 shows the average wear values recorded on the test plate when lubricating with calcium greases. For the base Sample C, the wear volume on the test plate was 50.724 × 10^4^ µm^3^ at a 50 N load, 98.291 × 10^4^ µm^3^ at a 100 N load, and 156.860 × 10^4^ µm^3^ at a 150 N load. These values are higher than those obtained in tests with the base lithium grease. Therefore, the importance of the base grease and its characteristics should be emphasized once again.

Wear was reduced when lubricants doped with hexagonal boron nitride were used. In this group of lubricants, additive A was also the most effective, which was related to it having the smallest particle size and highest nanoparticle content (Table 1). The lowest wear was recorded for the C_A3_ grease, with values of 15.212 × 10^4^ µm^3^ at a 50 N load, 29.292 × 10^4^ µm^3^ at a 100 N load, and 61.970 × 10^4^ µm^3^ at a 150 N load. This represents a reduction in wear of 70.0%, 70.2%, and 60.5%, respectively, compared to the base composition.

Hexagonal boron nitride, designated as B, was the second most effective additive in terms of anti-wear efficacy when introduced into calcium greases. Slightly higher wear was recorded with additive C compared to additive B. The relationships between the concentrations of additives A, B, and C were similar. Lubrication with compositions containing these additives at concentrations of 1% and 10% resulted in higher wear than that observed for the other two concentrations of hexagonal boron nitride.

Calcium greases containing additive D protected the mating surfaces from wear more effectively than the base composition. This improvement was more pronounced with lower concentrations of this type of hexagonal boron nitride in the lubricant. This observation is consistent with the findings regarding the anti-friction properties; however, their effect in this regard was relatively small compared to the other types of hexagonal boron nitride.

**Figure 7:**
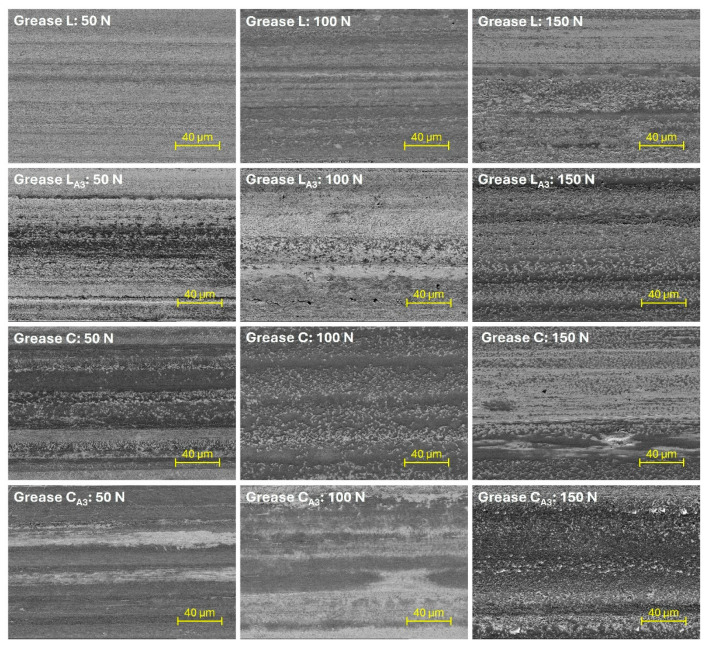
Surface images of wear marks on the test plate.

To evaluate how hexagonal boron nitride particles enhance the anti-friction and anti-wear performance of base greases, observations were made using a scanning electron microscope. Wear marks were analyzed for the base greases and greases with the best tribological properties, specifically the L_A3_ and C_A3_ compositions. Figure 7 shows images of the steel plate surface after friction processes under various applied loads. A microanalysis of the chemical composition was performed for both the untested components and wear marks formed on steel plates under a 150 N load. For both the analysis and imaging, the central region of these wear marks was examined.

When analyzing the results of the tribological tests performed under reciprocating motion, it was observed that wear on the plate increased with the applied load (Figure 5 and Figure 6). The most severe surface damage occurred at a load of 150 N. At lower loads, particularly at 50 N, the wear marks on the surface were smoother and showed less local damage (Figure 7). The increase in load likely caused more frequent local contact between the friction surfaces, characteristic of mixed friction. This resulted in more intense plate wear and greater surface damage. At a load of 50 N, the surface lubricated with base lithium grease appeared relatively smooth, with linear abrasions and some irregularities. In contrast, the surface lubricated with base calcium grease displayed more pronounced wear. As the load increased, surface damage worsened, with wear marks becoming more irregular, indicating increased lubricant film instability. This instability was further confirmed by the analysis of the friction resistance results (Figure 3 and Figure 4). The use of greases containing hexagonal boron nitride resulted in reduced wear of the steel plate (Figure 5 and Figure 6), likely due to the deposition of hexagonal boron nitride on the friction surfaces and its active effect. As a result, the appearance of surfaces lubricated with compositions containing h-BN differed from those treated with the base greases (Figure 7); the surfaces of the wear marks formed when lubricated with grease containing h-BN exhibited fewer defects, resulting in a more homogeneous structure compared to those lubricated with the base samples. At a load of 50 N, the surfaces treated with greases containing hexagonal boron nitride showed signs of wear but no distinct local damage. As the load increased, the surface wear progressed similarly to that with the base greases, but the surface structure still remained homogeneous. Although the condition of the surface lubricated with the calcium greases improved due to the hexagonal boron nitride particles, the use of this type of grease still resulted in more damage to the steel substrate than was observed with the lithium greases.

The observations of the surface condition of the steel plates were supplemented by an analysis of the chemical microcomposition of the surface of the wear marks formed on the steel plates under a 150 N load. The steel plate was fabricated in accordance with the standard [34]. In analyzing the microchemical composition of the surface, it was also necessary to consider the second component of the friction system used in the UNMT, which was a ball made of 100Cr6 steel in accordance with the standard [35]. Therefore, the microchemical composition of the surface of a selected ball and plate was determined prior to the tribological testing. The data obtained provided the foundation for subsequent analyses performed on surfaces treated with greases. The results are presented in Table 5.

Among the elements detected on the surfaces of the steel ball and steel plate, those typical of the chemical composition of this steel grade were observed. On the wear marks formed on the steel plate during lubrication with the base lithium grease and base calcium grease, in addition to elements characteristic of the friction pair components, oxygen was detected. Oxygen was present on all surfaces lubricated with the greases, attributed to the interactions between the greases and the steel substrate, leading to oxidation and the formation of oxides that could adhere to the surfaces of the analyzed components. Calcium was also detected in the C_A3_ grease. This is due to the presence of a thickener in the grease, which is a product of the reaction of fatty acids with calcium hydroxide. As a result of lubrication with the lithium and calcium greases containing hexagonal boron nitride, boron was detected on the surfaces of the wear marks formed on steel plates in addition to the components of the friction system. Thus, the active role of hexagonal boron nitride in the analyzed tribological systems—related to the protection of mating surfaces—was indicated.

## 4. Discussion

This study focused on the tribological properties of lithium- and calcium-based greases to which nanoparticles and microparticles of hexagonal boron nitride were added at various concentrations.

The tribological properties of greases were improved by the addition of hexagonal boron nitride to the base greases. This conclusion aligns with the findings of other studies cited in the introduction [15,18,23,24,25,26,27,28,29], although those studies focused on different base greases doped with hexagonal boron nitride, which exhibited varying physical and chemical properties.

The tribological properties of the greases were found to depend on the granulation of the hexagonal boron nitride particles. The use of smaller particle sizes resulted in lower coefficient of friction values and reduced wear (Figure 3, Figure 4, Figure 5 and Figure 6). The best results were recorded for additive A, which had the highest proportion of nanoparticles. This correlates with the findings presented in [28], which also compared the effects of nanoparticles and h-BN microparticles. Additionally, as previously shown in [33], greases containing smaller particles (additive A) demonstrated greater thermal and mechanical stability, which could also have contributed to their superior tribological performance. The low variability of the friction coefficient observed for the grease containing additive A under different load conditions indicates high operational stability, which is particularly desirable in industrial applications where mechanical loads are subject to fluctuation. This consistent performance suggests that additive A may be well suited for use in tribological systems requiring reliable lubrication under varying operating conditions.

The most appropriate particle concentration of additive A in terms of tribological performance was 3%. This was also the optimal concentration for additives B and C, which also contained nanoparticles (Table 1). At lower concentrations, such as 1%, the number of h-BN particles present in the contact zone was likely insufficient to form a continuous film capable of effectively reducing friction and wear. When the concentration was too low, the number of particles reaching the micro-contact regions was limited, resulting in incomplete surface coverage. Consequently, the beneficial effects of h-BN on the tribological properties of the grease were not fully realized. Excessive particle agglomeration could occur with these three additives, particularly at 10%. The formed agglomerates may have hindered the flow of grease to the microfriction areas, and they might not have reached these areas in the same way as larger particles. This resulted in lubrication instability during the test, as evidenced by variations in the coefficient of friction (Figure 3 and Figure 4). Consequently, this also negatively impacted the anti-wear performance of the greases.

The problem of particle agglomeration, particularly with hexagonal boron nitride nanoparticles, has been highlighted in previous studies. In [24], the negative effect of h-BN nanoparticle agglomeration on the tribological performance of greases was observed at a concentration of 0.75%, and in [26], this effect became especially pronounced above a concentration of 2%. A similar observation was made for a nanoparticle mixture consisting of 0.25% h-BN and 0.75% Al_2_O_3_ [27]. The different agglomeration thresholds reported in the cited studies may be attributed to variations in the base grease type, particle size, and dispersion behavior. For instance, in [24], h-BN particles with an average size of 449.35 nm were used in lithium grease, whereas in [26], much smaller particles (50 nm) were applied, also in lithium grease. In contrast, in [27], h-BN particles smaller than 100 nm were introduced into a calcium sulfonate complex grease. These differences in both particle size and base grease composition may have affected dispersion stability and, ultimately, the concentration at which agglomeration effects became dominant. However, based on an analysis of various publications on nanoparticles, the authors of [28] concluded that tribological performance decreases when concentrations exceed 4%. In this study, among the additives containing nanoparticles, the 3% concentration was found to be the most appropriate. Increasing the amount of hexagonal boron nitride to 5% and then to 10% resulted in a decrease in the tribological performance of the greases. Therefore, the observations of other studies regarding the concentrations of h-BN at which the agglomeration problem becomes significant are not entirely consistent with those found in this study. This discrepancy is likely due to the specifics of the tribological tests conducted and the characteristics of the grease, including the type of base grease and the physicochemical properties of the hexagonal boron nitride. This highlights the importance of correctly identifying these factors.

The tendency of nanoparticles to agglomerate results from the large surface area of their interfaces. In the case of nanopowders containing hexagonal boron nitride, this refers to the specific surface area [38]. Nanoparticles tend to agglomerate, particularly in lubricating oils; however, this problem is less severe in greases, which have a higher viscosity than lubricating oils, thereby reducing Brownian motion [29]. Additives A, B, and C, for which the possibility of particle agglomeration is noted, were characterized by a developed porous structure (Table 1). This suggests that the phenomenon of particle agglomeration occurred in these cases. This problem became particularly significant when an excessively high concentration of h-BN was used, specifically 10%.

The situation was different for additive D, which contained only microparticles. The lower the concentration of this additive, the lower the frictional resistance and wear of the friction system components (Figure 3, Figure 4, Figure 5 and Figure 6). This additive had the highest granularity, with an average particle size of 6985 nm (Table 1). A large number of these particles dispersed in lithium- or calcium-based greases resulted in an increase in frictional resistance. This could be attributed to both the high frictional resistance caused by the lamellar structure of the particles [32] and the aforementioned particle sizes, which affected the structure and rheological properties of the base material. The size of these particles meant that they reached the contact zone much less effectively than smaller particles and, therefore, provided less wear protection. Like agglomerates, they may also have impeded the flow of grease into the friction micro-regions.

The type of base grease and the interaction of the hexagonal boron nitride particles with the spatial structure of the grease played a significant role in shaping the observed tribological properties. As noted earlier, the influence of these factors is not explicitly described in the literature. In the case of the calcium greases, the friction coefficient values were higher due to their more compact consistency (lithium grease, NLGI class 2; calcium grease, class 4, which was investigated in [33]) and lower fluidity. For this reason, the lowest average coefficient of friction values were observed for the calcium grease samples at a load of 100 N (Figure 4a). This was true for the greases containing additive D as well as for the greases blended with the other three additives at concentrations of 1% or 10%. At higher mechanical loads (greater than 50 N), the calcium grease became more fluid, resulting in the more efficient distribution of the selected h-BN particles to the friction zone. This increased fluidity may also have been caused by a temperature rise in the friction zone, although this aspect was not analyzed in this study. The effect of load on the tribological properties of greases appeared to be strongly influenced by the type of base grease.

**Figure 8:**
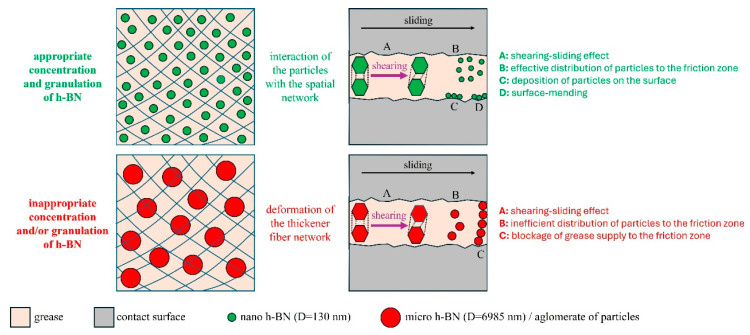
Mechanism of tribological interaction of hexagonal boron nitride particles in greases.

The settling of h-BN particles into the structure of the base greases was also an important factor. Smaller particles exhibited greater ease of integration into the spatial network of the greases, as demonstrated by the microstructural analysis of the greases described in [33]. These particles were readily deposited in the oil matrix and on the soap fibers without disrupting their network. As a result, during the lubrication process, they could effectively reach the friction zone, where they initiated mechanisms that reduced frictional resistance and wear of the cooperating elements (Figure 3, Figure 4, Figure 5 and Figure 6). Better dispersion of such particles was observed in the lithium grease, in which the larger spaces between the thickener fibers allowed particles of suitable granulation to settle. In contrast, the dispersion of additive D particles in the grease was associated with the deformation of the soap fiber network. It is reasonable to assume that this deformation increased with the concentration of this additive, which explains the deterioration in tribological performance as the concentration of additive D increased (Figure 3, Figure 4, Figure 5 and Figure 6). The deformation of the network could also have been caused by the agglomeration of smaller particles, including nanoparticles with an extended porous structure, when applied at too high a concentration. This was confirmed by the results obtained for additives A, B, and C at a concentration of 10% (Figure 5, Figure 6, Figure 7 and Figure 8).

The proposed mechanism of the anti-friction and anti-wear action of hexagonal boron nitride particles, based on the results of the tests conducted in this study, is shown in Figure 8. The presence of hexagonal boron nitride in the wear tracks was confirmed by the microanalysis of the surface’s chemical composition. Therefore, it can be concluded that the hexagonal boron nitride particles penetrated the friction zone along with the grease, subsequently depositing on the surface and forming protective layers. The individual layers of h-BN consist of boron and nitrogen atoms connected by strong covalent bonds. Weak intermolecular interactions between adjacent layers, categorized as van der Waals forces, make them easily sheared. These layers are arranged parallel to the direction of sliding movement, thereby reducing frictional resistance. This lubrication mechanism, known as the shearing-sliding effect, has been attributed to nanoparticles of layered materials [39]. However, this applies to both hexagonal boron nitride nanoparticles and microparticles due to the crystal structure of this material.

Smaller particles, particularly hexagonal boron nitride nanoparticles, characterized by their expanded porous structure, were able to penetrate more easily into the contact area of the cooperating surfaces. The surfaces (Figure 7) lubricated with the 3% hexagonal boron nitride compositions from Sample A, which contained the most nanoparticles (Table 1), showed less local surface damage than those lubricated with the base compositions. This suggests that the aforementioned h-BN particles may have been deposited in areas of material loss on the friction surfaces. This reduced contact between the asperities, leading to a decrease in frictional resistance and wear of the mating surfaces, as reported in the earlier sections. Such an effect is attributed to nanoparticles and is commonly referred to as surface-mending [13,39,40,41,42].

As in the present case, the anti-wear and anti-friction effects of hexagonal boron nitride have previously been explained by their deposition or adsorption onto the surface, forming a protective layer [23,24]. This results in an improved surface condition compared to lubrication with base compositions [20,23,24,25,26,27,28,29]. The greater effectiveness in this regard is attributed to hexagonal boron nitride nanoparticles, which, due to their size, can more easily reach the contact zone compared to microparticles [28], as confirmed in this study. The rolling effect achieved when lubricating with hexagonal boron nitride nanoparticles has also been reported in the literature [23,29]. This lubrication mode is characteristic of nanoparticles and involves particles with a spherical or quasi-spherical shape. This effect is associated with low loading conditions as it allows the shape and stiffness of the nanoparticles to be maintained [13,39,40,41,42]. However, the hexagonal form is a soft variant of boron nitride. The micrographs of the particles used in this study, which are reported in [32], show that the h-BN particles were not spherical. Additionally, the loads applied in this study resulted in significant initial unit pressures of approximately 3.7–5.3 GPa. Given the flake-like morphology and high contact pressures, the rolling effect is less likely, although local rotation under shear cannot be ruled out.

## 5. Conclusions

In this study, the anti-friction and anti-wear properties of lithium and calcium greases doped with nanoparticles and microparticles of hexagonal boron nitride were evaluated. Tribological tests were carried out on a ball-on-flat system in reciprocating motion at three different loads. The lubricating efficiency of hexagonal boron nitride nanoparticles and microparticles under these conditions was evaluated in relation to the type of base grease. Hexagonal boron nitride was found to be an effective additive for improving the tribological performance of lithium- and calcium-based greases. The anti-friction and anti-wear properties of the doped greases were determined by the concentration and granulation of the hexagonal boron nitride particles as well as the type of base grease. The following conclusions can be drawn:

1. In the group of lithium greases, the best anti-friction properties were achieved with a composition containing 3% h-BN, with an average particle size of 130 nm and the highest proportion of nanoparticles. The average coefficient of friction for this composition was 4.4–9.7% lower than that of the base grease, depending on the applied load. Additionally, grease with this composition reduced the wear of the samples by 66.9–69.2% compared to the base grease.
2. The best anti-friction properties among the calcium greases were achieved with a 3% h-BN blended composition, with an average particle size of 130 nm and the highest proportion of nanoparticles. Compared to the calcium base grease, the use of this composition resulted in a 13.9–18.2% reduction in frictional resistance, depending on the applied load. The anti-wear performance of this calcium grease was also the best, as confirmed by the 60.5–70.2% reduction in wear values at the set loads.
3. The differences in the tribological properties observed between the lithium and calcium greases were due to their various spatial structures and associated functional properties, such as consistency, thermal stability, and mechanical stability. The type of base grease influenced how the grease responded to changes in nodal load, which directly affected their tribological properties under different operating conditions.
4. The tribological performance of the evaluated compositions was determined by the particle dispersion in the base grease, which was influenced by the arrangement and fiber size of the thickener as well as the particle morphology of the h-BN. The additive particles with the smallest average particle size (130 nm), the highest proportion of nanoparticles, and an extended porous structure were best integrated into the base grease. These particles effectively penetrated the friction zone along with the grease, initiating mechanisms that reduced the frictional resistance and wear of the friction system components.

It should be noted that all the tests were conducted at ambient temperature under controlled laboratory conditions and involved a limited number of grease formulations. These factors should be considered when interpreting the results and evaluating their relevance to real-world operating environments.

## Figures and Tables

**Table 1:** Physicochemical properties of hexagonal boron nitride [32].

Additive Sample	Apparent Diameter D [nm]	Part of Nanoparticles [%]	Specific Surface Area S_BET_ [m^2^·g^−1^]	Total Pore Volume V_t_ [cm^3^·g^−1^]	Typical Pore Size [nm]
A	130	33.4	30	0.15	0.5; 3.5; 23.0
B	188	10.9	33	0.20	3.8; 23.0
C	193	9.2	30	0.16	3.9; 23.0
D	6985	0.0	7	0.04	4.3; 24.0

**Table 2:** Summary of test samples.

Additive Sample	Lithium Grease (L)				Calcium Grease (C)			
	Concentration of Hexagonal Boron Nitride (wt. %)							
	1	3	5	10	1	3	5	10
A	L_A1_	L_A3_	L_A5_	L_A10_	C_A1_	C_A3_	C_A5_	C_A10_
B	L_B1_	L_B3_	L_B5_	L_B10_	C_B1_	C_B3_	C_B5_	C_B10_
C	L_C1_	L_C3_	L_C5_	L_C10_	C_C1_	C_C3_	C_C5_	C_C10_
D	L_D1_	L_D3_	L_D5_	L_D10_	C_D1_	C_D3_	C_D5_	C_D10_

**Table 3:** Chemical composition of steel balls and plates [34,35].

Element	Steel Balls	Stell Plates
C	0.93–1.05	0.95–1.05
Mn	0.25–0.45	0.25–0.45
Si	0.15–0.35	0.17–0.37
P	≤0.025	≤0.027
S	≤0.015	≤0.020
Cr	1.35–1.60	1.30–1.65
Cu	≤0.30	≤0.025
O	≤0.0015	≤0.002
Mo	≤0.10	-
Al	≤0.050	-
Ni	-	≤0.030
Ti	-	≤0.01

**Table 4:** UNMT reciprocating test conditions.

Parameter	Value		
Ball radius [mm]	3.2		
Stroke length [mm]	10		
Load [N]	50	100	150
Initial contact pressures [MPa]	3691.17	4650.59	5323.60
Oscillating frequency [Hz]	10		
Test duration [s]	2000		
Sliding distance [m]	400		

**Table 5:** Mass fraction of elements detected on the surface of friction components.

Element	Ball	Flat	Grease L	Grease L_A3_	Grease C	Grease C_A3_
Fe	97.87	97.69	93.75	79.97	96.31	80.40
Cr	1.54	1.68	1.70	1.52	1.59	1.43
Mn	0.34	0.36	0.31	0.29	0.42	0.29
Si	0.25	0.27	0.27	0.26	0.27	0.26
O	-	-	3.97	6.82	1.41	7.41
Ca	-	-	-	-	-	1.05
B	-	-	-	11.14	-	9.16

## Data Availability

The original contributions presented in the study are included in the article, further inquiries can be directed to the corresponding author.
